# FAK and S6K1 Inhibitor, Neferine, Dually Induces Autophagy and Apoptosis in Human Neuroblastoma Cells

**DOI:** 10.3390/molecules23123110

**Published:** 2018-11-28

**Authors:** Dinh-Chuong Pham, Yu-Chuan Chang, Shian-Ren Lin, Yuh-Ming Fuh, May-Jywan Tsai, Ching-Feng Weng

**Affiliations:** 1 Faculty of Applied Sciences, Ton Duc Thang University, Ho Chi Minh City 700000, Vietnam; phamdinhchuong@tdtu.edu.vn; 2 Department of Life Science and Institute of Biotechnology, National Dong Hwa University, Shoufeng, Hualien 97401, Taiwan; kevin7699402@hotmail.com (Y.-C.C.); mecurry@gmail.com (S.-R.L.); ming56564542@yahoo.com.tw (Y.-M.F.); 3 Neural Regeneration Laboratory, Taipei Veterans General Hospital, Taipei 11260, Taiwan; mjtsai2@vghtpe.gov.tw

**Keywords:** neferine, FAK/S6K1, autophagy, apoptosis, human neuroblastoma cells

## Abstract

Human neuroblastoma cancer is the most typical extracranial solid tumor. Yet, new remedial treatment therapies are demanded to overcome its sluggish survival rate. Neferine, isolated from the lotus embryos, inhibits the proliferation of various cancer cells. This study aimed to evaluate the anti-cancer activity of neferine in IMR32 human neuroblastoma cells and to expose the concealable molecular mechanisms. IMR32 cells were treated with different concentrations of neferine, followed by 3-(4,5-dimethylthiazol-2-yl)-2,5-diphenyltetrazolium bromide (MTT) assay to assess cell viability. In an effort to determine the molecular mechanisms in neferine-incubated IMR32 cells, cell cycle arrest, cell migration, and focal adhesion kinase (FAK), the 70-kDa ribosomal S6 kinase 1 (S6K1), poly (ADP-ribose) polymerase (PARP), caspase-3, Beclin-1, and microtubule-associated protein 1A/1B-light chain 3 (LC3) protein expressions were investigated. Neferine strongly disrupted the neuroblastoma cell growth via induction of G2/M phase arrest. Furthermore, neferine provoked autophagy and apoptosis in IMR32 cells, confirmed by p-FAK, and p-S6K1 reduction, LC3-II accumulation, Beclin-1 overexpression, and cleaved caspase-3/PARP improvement. Finally, neferine markedly retarded cell migration of neuroblastoma cancer cells. As a result, our findings for the first time showed an explicit anti-cancer effect of neferine in IMR32 cells, suggesting that neferine might be a potential candidate against human neuroblastoma cells to improve clinical outcomes with further in vivo investigation.

## 1. Introduction

Neuroblastoma (NB) is the most common malignant nervous system tumor in babies and the third-most common cancer in children, accounting for ~8% of childhood cancers and 15% of all pediatric cancer deaths [1]. Even though some newborns can recuperate spontaneously, many of those damaged have poor prognosis and aggressive tendency of metastasis [2]. Ongoing treatment used in the administration of neuroblastoma includes surgery, chemotherapy, radiotherapy, and biotherapy. In spite of rigorous multi-combining therapy, the overall survival rates are less than 40% of the cases [3]. Some chemo drugs, like cyclophosphamide, doxorubicin, and etoposide show low potency with numerous side effects because the low penetrations of blood brain barrier (BBB) resulted in low bioavailability. It has been shown that neuroblastoma gets resistance to cytotoxic drugs due to stable genetic alterations occurring during treatment [4]. Therefore, effective and safe drugs are high-priority required.

Nature is the best supplier of natural drugs where more than 600 approved anti-cancer products were isolated [5]. Neferine is a prime bisbenzylisoquinoline alkaloid, obtained from the green seed embryos of the lotus *Nelumbo nucifera* [6]. Previous works have proved that neferine effectively inhibits the proliferation of multidrug-resistant cancer cells [7], induces autophagy in lung cancer cells [8], regulates apoptosis in HSC-T6 cells [9], and enhances the anti-tumor activity of chemo drugs like cisplatin [10], and doxorubicin [11]. Recently, our research group has shown that neferine is a novel dual inhibitor of focal adhesion kinase (FAK) and the 70-kDa ribosomal S6 kinase 1 (S6K1) via molecular docking [12]. FAK and S6K1 proteins are the important candidate targets against which anticancer treatments could be developed. Although neferine is tested on various types of cancer, no particular study has been described its activity on human neuroblastoma tumor cells. In this study, human neuroblastoma tumor cells-IMR32 cells were treated with various concentrations of neferine, followed by MTT assay to measure cell viability. In an effort was further to investigate the molecular mechanisms of neferine-incubated IMR32 cells through cell cycle arrest, cell migration, and FAK, S6K1, PARP, caspase-3, Beclin-1, and LC3 protein expressions. Temozolomide, a clinical reagent of brain tumors, which can induce autophagy or apoptosis signaling pathways in malignant glioma cells [13,14,15], was used as a positive control of anti-cancer activity in this study. Herein, this is first evidenced that neferine induces autophagy and apoptosis in IMR32 human neuroblastoma cells through down-regulation of FAK and S6K1 pathways.

## 2. Results

### 2.1. Neferine Suppresses Cell Proliferation in Human Neuroblastoma Cells

**Figure 1:**
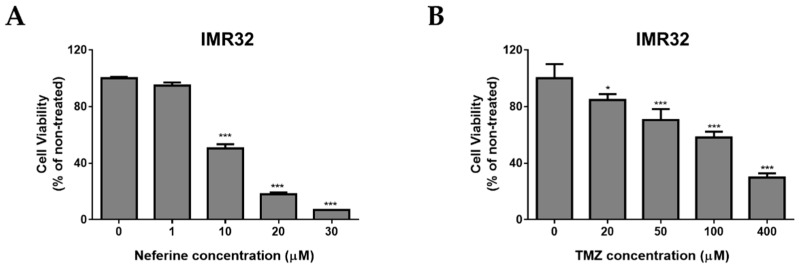

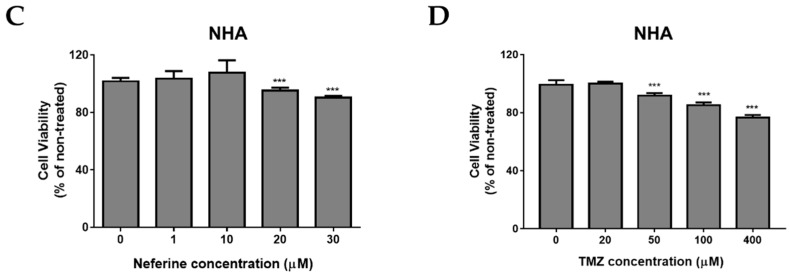
Neferine suppresses cell proliferation in human neuroblastoma cells. (**A**,**B**) IMR32 cells were treated with 1, 10, 20, and 30 μM of neferine or 20, 50, 100, and 400 μM of TMZ for 24 h; (**C**,**D**) Normal human astrocytes (NHA) were exposed to the indicated doses of neferine and TMZ for 24 h. Cell viability was analyzed by MTT assay, and the surviving cells were determined and presented as a percentage of the non-treated cells. Data are presented as mean ± standard deviation (SD) in three independent experiments. * *p* < 0.05, *** *p* < 0.001 as compared with the non-treated control.

In order to determine the cytotoxicity effects of neferine on IMR32 human neuroblastoma cell line, the cells were cultured and treated with various concentrations of neferine or temozolomide (TMZ), respectively for 24 h (Figure 1), followed by using MTT assay to analyze the cell viability. As expected, neferine significantly induced IMR32 cell death in a dose-dependent manner with IC50 (the half maximal inhibitory concentration) at 10 μM for 24 h (*p* < 0.001, Figure 1A). However, IMR32 cells were much less susceptible to TMZ, exhibiting an IC50 at 191 μM for 24 h (*p* < 0.001, Figure 1B). Next, we determined the cytotoxic effects of neferine on normal human astrocytes in comparison with TMZ. As shown in Figure 1C, neferine treatment exhibited much less cytotoxicity (<10%, *p* < 0.001) at dose 30 µM for 24 h incubation in normal astrocytes. The cytotoxicity of neferine for the normal cells showed much lower levels than for the neuroblastoma cells tested under the same conditions. TMZ treatment induced higher levels of cytotoxicity (<25%, *p* < 0.001) at dose 400 µM for 24 h incubation in normal human astrocytes (Figure 1D). These results indicate that neferine induces tumor cell-specific proliferation-inhibiting activity at low concentrations. 

### 2.2. Neferine Induces G2/M Cell Cycle Arrest in Human Neuroblastoma Cells

**Figure 2:**
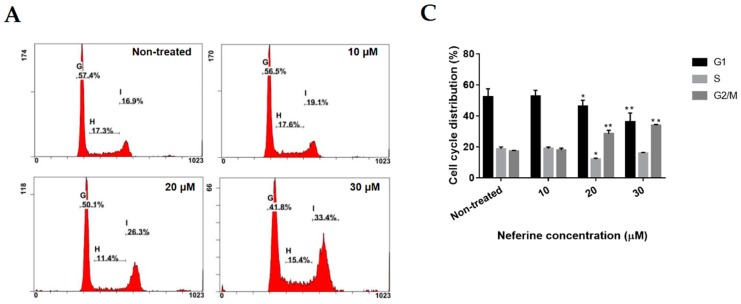

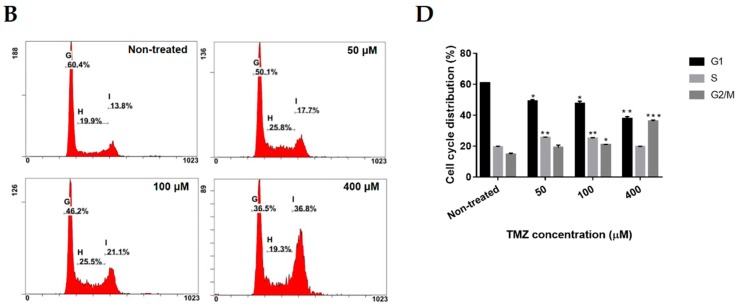
Neferine induces G2/M cell cycle arrest in human neuroblastoma cells. (**A**,**B**) IMR32 cells were cultured at the described concentrations of neferine (**A**) or TMZ (**B**) for 24 h, and then flow cytometry analysis was used to determine the cell cycle distributions; (**C**,**D**) The quantification of cell cycle arrest in neferine-treated or TMZ-treated IMR32 cells was shown. Data are expressed as mean ± SD in three independent experiments. * *p* < 0.05, ** *p* < 0.01, *** *p* < 0.001 as compared with the non-treated control.

To check if the cell growth inhibition is related to cell cycle arrest, we measured the role of neferine in the cell cycle distribution. IMR32 cells were treated with the indicated concentrations of neferine or TMZ for 24 h, and then analyzed using PI method. As shown in Figure 2, the percentage of IMR32 cells incubated with 30 μM neferine (Figure 2A,C) or 400 μM TMZ (Figure 2B,D) at G1/S phase was strongly decreased from 70.9% and 79.7% to 51.4% and 58.7%, respectively (*p* < 0.01), while the proportion of neuroblastoma cells at G2/M phase was strikingly increased from 17.3% and 14.6% to 33.9% and 35.95%, respectively (*p* < 0.001). Therefore, the data manifested that low-dose neferine caused G2/M cell cycle arrest in IMR32 neuroblastoma cells after 24 h treatment.

### 2.3. Neferine Inhibits p-FAK and p-S6K1 Protein Levels in Human Neuroblastoma Cells

**Figure 3:**
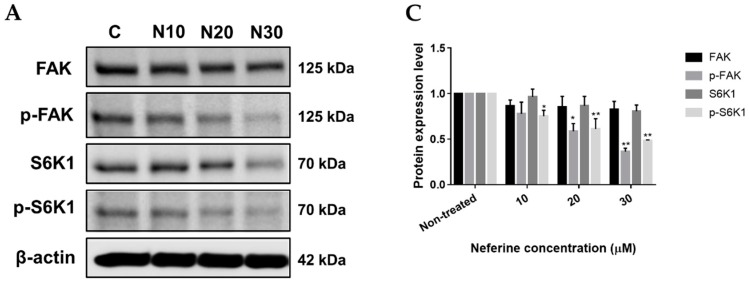

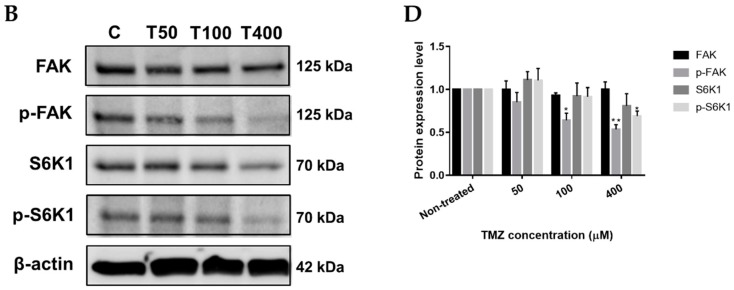
Neferine inhibits FAK, p-FAK, S6K1, and p-S6K1 protein levels in human neuroblastoma cells. (**A**,**B**) IMR32 cells were incubated with the desired concentrations of neferine (**A**) or TMZ (**B**) for 24 h. Then, all cells were harvested and lysed for Western blot analysis; (**C**,**D**) Changes in the levels of FAK, p-FAK, S6K1, and p-S6K1 proteins after being normalized to the levels of beta actin were presented. Data are shown as mean ± SD of three independent experiments. * *p* < 0.05, ** *p* < 0.01 as compared with the non-treated control.

FAK and S6K1 are well-known targets for anti-cancer therapy because of their key roles in cell proliferation, cell viability, and cell migration [16,17]. To determine the effects of neferine on FAK, p-FAK, S6K1, and p-S6K1 proteins, IMR32 cells were incubated with different concentrations of neferine or TMZ, respectively for 24 h, and Western blot analysis was used to measure protein levels. In IMR32 cells treated with 30 μM neferine, the protein levels of p-FAK and p-S6K1 were significantly reduced by approximately 3.2-fold and 2.1-fold than those in non-treated control, respectively (*p* < 0.01, Figure 3A,C). In keeping with the detection acquired in the IMR32-incubated neferine, treatment of IMR32 cells with 400 μM TMZ also inhibited the p-FAK and p-S6K1 protein expression levels by 2-fold and 1.5-fold than those in non-treated control, respectively (*p* < 0.01, Figure 3B,D). The protein levels of FAK and S6K1 were not significantly different in neferine or TMZ-treated cells compared with non-treated control. Thus, the results revealed that neuroblastoma cell proliferation was obstructed by the loss of p-FAK and p-S6K1 proteins under neferine treatment.

### 2.4. Neferine triggers autophagy in human neuroblastoma cells

**Figure 4:**
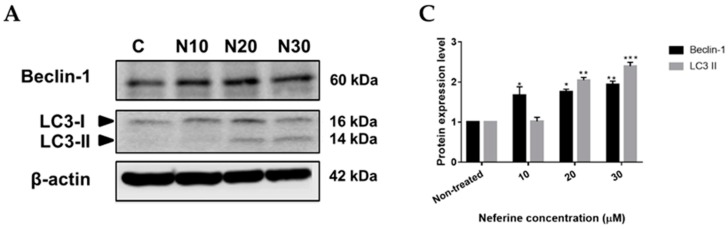

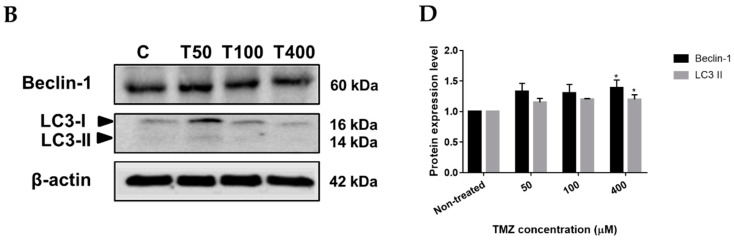
Neferine triggers autophagy in human neuroblastoma cells. (**A**,**B**) IMR32 cells were exposed to the indicated concentrations of neferine (**A**) or TMZ (**B**) for 24 h. Then, autophagy-related proteins, including Beclin-1 and LC3, were assessed using Western blot analysis; (**C**,**D**) Changes in the levels of Beclin-1, LC3-I, and LC3-II after being normalized to the levels of beta actin were shown. The data are expressed as mean ± SD in three independent experiments. * *p* < 0.05, ** *p* < 0.01, *** *p* < 0.001 as compared with the non-treated control.

Autophagy is the natural, catabolic procedure forming autophagosomes, the double-membrane vesicles, to uptake cytoplasmic content, which is degraded and recycled by fusion of the autophagosomes and the lysosomes [18]. Augmentation of autophagy-dependent cell death from abnormal cells is one of the perfect choices among anti-cancer therapies [8]. To check whether neferine could induce autophagy in neuroblastoma cells or not, IMR32 cells were treated with various concentrations of neferine or TMZ, respectively for 24 h, followed by Western blot analysis. Microtubule-associated protein 1A/1B light chain 3-II (LC3-II) and Beclin-1 are the valid autophagosomal markers to reflect autophagic cell death and autophagy formation [19,20]. Beclin-1 expression and LC3-II conversion were found to be strongly induced by neferine incubation in a dose-dependent manner, especially with 2-fold and 2.4-fold higher than those of non-treated control, respectively (*p* < 0.01, Figure 4A,C) at 30 μM neferine for 24 h. TMZ showed similar induction on Beclin-1 and LC3-II formation, although to a lesser extent than that of neferine (*p* < 0.05, Figure 4B,D). Taken together, the data conveyed that neferine could trigger autophagy in human neuroblastoma cells.

### 2.5. Neferine Induces Apoptosis in Human Neuroblastoma Cells

**Figure 5:**
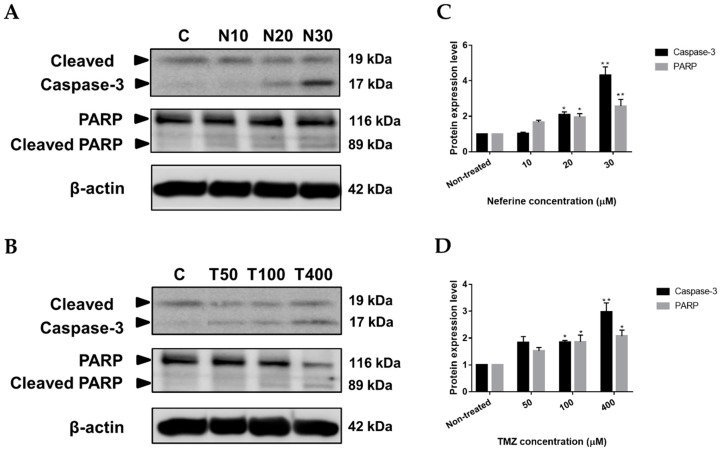
Neferine induces apoptosis in human neuroblastoma cells. (**A**,**B**) IMR32 cells were incubated at the described concentrations of neferine (**A**) or TMZ (**B**) for 24 h. Then, apoptosis-related proteins, including cleaved Caspase-3 and PARP, were determined using Western blot analysis; (**C**,**D**) Changes in the levels of cleaved Caspase-3 and PARP after being normalized to the levels of beta actin were displayed. Data are shown as mean ± SD in three independent experiments. * *p* < 0.05, ** *p* < 0.01 as compared with the non-treated control.

Apoptosis is a typical and crucial mode of programmed cell death, which involves the elimination of cells by two well-established mechanisms, the intrinsic and extrinsic pathways [21]. Cell death by anti-cancer drugs are closely related to pivotal molecular mechanisms of apoptosis. As a consequence, if apoptosis correlated with the growth inhibition of human neuroblastoma cells by neferine was further investigated. It is remarkable that caspase-3 and PARP cleavage are known apoptotic markers. The Western blot outcome showed that cleaved Caspase-3 and PARP were greatly elevated by neferine (*p* < 0.01, Figure 5A,C) or TMZ (*p* < 0.05, Figure 5B,D) culture in a dose-dependent manner. Hence, the results proved that IMR32 neuroblastoma cell suppression was associated with apoptosis caused by neferine administration. 

### 2.6. Neferine Inhibits Migration in Human Neuroblastoma Cells

**Figure 6:**
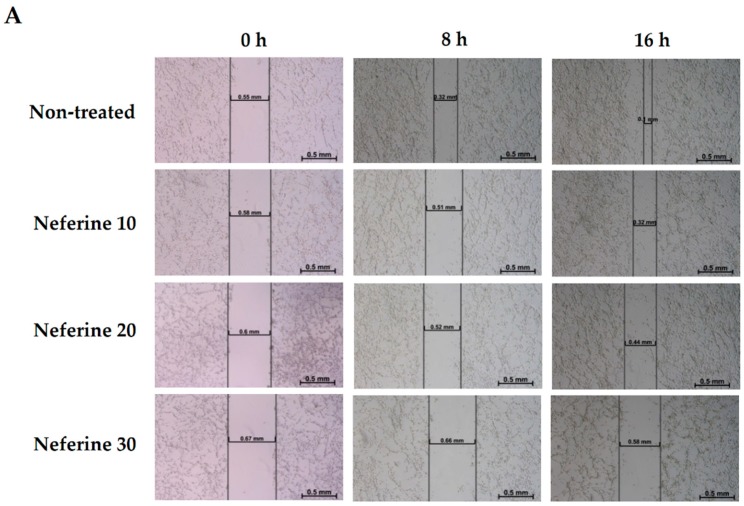

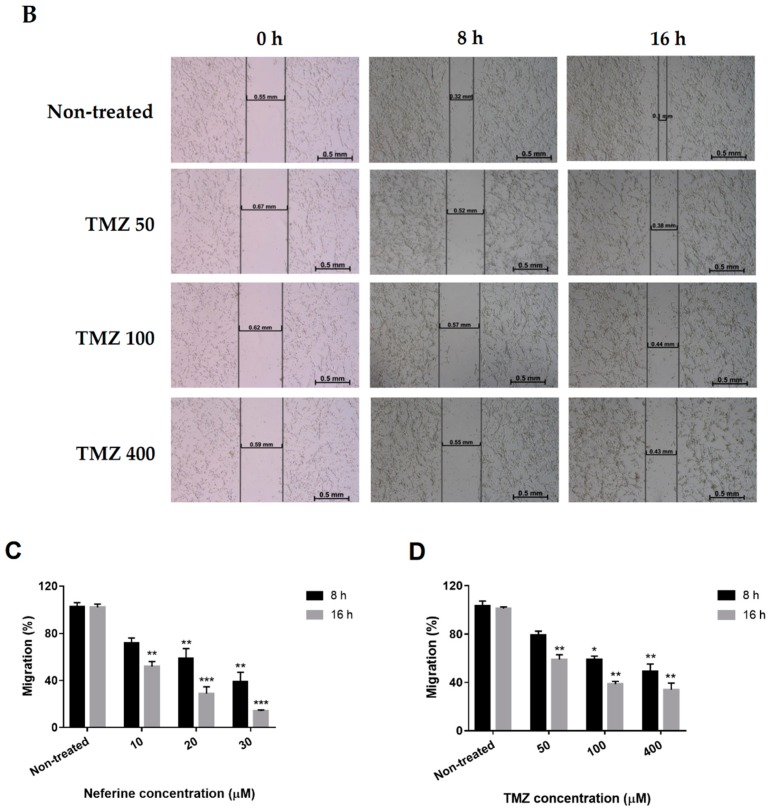
Neferine inhibits migration in human neuroblastoma cells. (**A**,**B**) IMR32 cell monolayer was scratched and treated with neferine (**A**) or TMZ (**B**) in time-dependent manner for wound-healing migration assay. Images were accessed by inverted microscope; (**C**,**D**) The quantification of cell migration in wound healing assay after treatment with neferine (**C**) or TMZ (**D**) were measured. Data are shown as percentages of the recovered scratch area relative to non-treated control. Results are presented as mean ± SD in three independent experiments. * *p* < 0.05, ** *p* < 0.01, and *** *p* < 0.001 as compared with the non-treated control.

Tumor metastasis is a significant factor which gives rise to cancer-related mortalities. The expanding migration assists cancer cells in underlining tumor invasion [22]. For that reason, inhibiting cancer cell migration could be prospective target for chemotherapy. Therefore, the potential anti-migration effect of neferine or TMZ on IMR32 cells was carried out through a wound healing assay. As supposed, neferine (Figure 6A) and TMZ (Figure 6B) remarkably suppressed neuroblastoma cell motility in time- and dose-dependent manners when compared with that of the non-treated control (*p* < 0.01). The wound areas of 30 μM neferine and 400 μM TMZ treatments were 85.4% and 67.5%, respectively (Figure 6C,D). These results demonstrated that neferine displayed a better migration inhibition activity than TMZ on human neuroblastoma cells.

## 3. Discussion

In this decade, numerous common foods provide potential benefits for the anti-cancer remedy, such as soy beans, green tea, and red wine [23,24]. *Nelumbo nucifera*, known as lotus, is spread all over the world and all the segments of lotus plant are consumable. Evidence has proved that the green seed embryos of lotus plant are important for pharmaceutical. Neferine is a bis-benzylisoquinoline alkaloid, which is isolated from seed embryos of lotus plant, has been shown for its inhibitory effects on the proliferation of liver cancer cells, lung cancer cells, and osteosarcoma cells [8,25,26]. Because of the great potentials for treating different types of cancer, neferine was a potential candidate in this study to further unveil its anti-cancer capacity. To the best of our knowledge, this is the first report to investigate neferine activity in human neuroblastoma cells. Here, we discovered that neferine could retard cell growth, induce G2/M cell cycle arrest, decrease FAK, p-FAK, S6K1, and p-S6K1 protein levels, cause autophagy and apoptosis, and block tumor cell migration in human IMR32 neuroblastoma cells.

Cell cycle arrest plays an important role to maintain genome stability because it allows cells with DNA damage to repair of mutations [27]. Decay of cell cycle arrest leads to cancer progression [28]. Up till now, many natural compounds were reported to possess malignant growth inhibition activity by blocking the cell cycle [29,30,31]. Importantly, cell cycle arrest at G2/M phase might be the key to cause the cancer cell death [32,33]. The results showed that neferine strongly retarded IMR32 cell proliferation in dose-dependent fashion (*p* < 0.001, Figure 1) and this reduction in cell growth arose from G2/M cell cycle arrest. After 24 h of neferine treatment, the G2/M cell population was dramatically increased at the expense of a decreased G1/S cell population of IMR32 neuroblastoma cells (*p* < 0.01, Figure 2).

Focal adhesion kinase (FAK), a 125 kDa non-receptor protein tyrosine kinase, has been shown to be important for survival signaling, motility, and metastasis and is overexpressed in a number of tumors [34]. FAK has been implicated in the control of cell proliferation where it is thought to mediate signals from growth factor receptors and integrins to regulate cell cycle progression [35]. It has been shown that FAK regulates proliferation of cancer cells [36] and the silencing of FAK inhibits cell proliferation in gastric cancer and lung cancer [37,38]. In addition, the 70-kDa Ribosomal S6 kinase 1 (S6K1), a 70 kDa Ser/Thr protein kinase, has been shown to control the cell proliferation [39]. In recent years, S6K1 was found to be overexpressed in brain tumors [40], breast tumors [41], and S6K1 protein expression has been according to the poor prognosis of those cancers. Thus, the targeting of FAK together with S6K1 may give a powerful approach for the treatment of cancer patients. Our previous study showed that some natural compounds, such as neferine, antroquinonol D, and curcumin could bind to FAK and S6K1 demonstrated by molecular docking method, and then inhibit C6 rat glioma cell proliferation [12]. Furthermore, our results revealed that neferine combined with cisplatin could down regulate the expression of Bcl-2, up regulate the expression of Bax, Bad, Bak, release of cytochrome c, p53 levels, activate cleavage forms of caspase-9, caspase-3, and PARP, and reduce the protein levels of FAK and VEGF [42]. In the present study, we proved that neferine treatment dramatically decreased the protein levels of p-FAK and p-S6K1 in IMR32 human neuroblastoma cells. The protein expression levels of p-FAK and p-S6K1 in 30 µM of neferine were much lower than those in 400 µM of TMZ administration (*p* < 0.01, Figure 3).

Autophagy is type II programmed cell death which responses to various anti-cancer therapies in many kinds of tumors [43]. The fusion between autophagosome and lysosome leading to the degradation of targeted cytoplasmic constituents is a key mechanism of autophagy [44]. Beclin-1 and microtubule-associated protein 1A/1B light chain 3 (LC3) play the crucial roles in autophagy pathway. LC3-II, known as autophagosomal marker, increases from the conjugation between LC3-I and phosphatidyl ethanolamine when autophagic cell death is activated [45,46]. Besides, overexpression of Beclin-1, the mammalian ortholog of yeast Atg6, results in the elevation of autophagic cell death [47,48]. In this study, neferine induced autophagy in dose-dependent manner, which the enhancement of LC3-I/LC3-II and Beclin-1 (*p* < 0.01, Figure 4). Our data are congenial with the previous findings of Cheng and colleagues, 2017 who showed that FAK was a novel negative regulator of Beclin1-mediated autophagy [49].

Apoptosis is a highly regulated process which leads to cellular morphological changes and cell death. Induction of apoptosis is a useful vehicle of targeted treatment in cancer [50]. Execution phase regarded as the terminal pathway of apoptosis, can activate the prosecution caspases, such as caspase-3, caspase-6 to cleave distinct substrates including cytokeratins, PARP to undergo apoptosis [51]. Therefore, cleaved caspase-3 and cleaved PARP are recognized as markers of apoptosis [52]. Previous researches also indicated that FAK depletion enhanced susceptibility to DOX-induced myocyte apoptosis and cardiac dysfunction [53,54]. We found that caspase-3 and PARP were remarkably cleaved with an increased concentration of neferine administration (*p* < 0.01, Figure 5).

It is notable that cancers lack angiogenesis standing reposefully. Suppressing angiogenesis might play a role in anti-cancer therapy to inhibit tumor progression and diminish the threat of metastasis [55]. Our results showed that neferine crucially reduced migration in IMR32 human neuroblastoma cells (*p* < 0.01, Figure 6). In this study, TMZ as a positive control could arrest the cell cycle of IMR32 cells at G2/M phase, increase the levels of apoptotic proteins, cleaved caspase-3 and cleaved PARP, as well as the levels of autophagy-related proteins, Beclin-1 and LC3-II, and retard IMR32 cell migration. As compared with the reference drug TMZ, neferine as a potent anti-brain tumor through the causes of apoptosis and autophagy as TMZ is demonstrated and further supported the first evidence for the suppression of FAK and S6K1 proteins as an inhibitor.

## 4. Materials and Methods 

### 4.1. Chemicals and Antibodies

Neferine was isolated according to the previous method [6]. Temozolomide was obtained from Orion Corporation (New Jersey, USA). The primary antibodies of FAK, p-FAK, S6K, p-S6K, Beclin-1, LC3, Cleaved Caspase-3, and PARP were purchased from Cell Signaling (Danvers, MA, USA). The antibody for β-actin was obtained from Sigma (Kawasaki, Japan), the anti-mouse and anti-rabbit IgG horseradish peroxidase-conjugated secondary antibodies were purchased from GE Healthcare (Chicago, IL, USA).

### 4.2. Cell Culture

IMR32 human neuroblastoma cell line was provided by Dr. May-Jywan Tsai (Neural Regeneration Laboratory, Taipei Veterans General Hospital, Taipei, Taiwan). IMR32 cells were maintained in DMEM (low glucose, pH 7.4) supplemented with 10% fetal bovine serum (FBS, Gibco, MA, USA), 2 mM L-glutamine, 1% NEAA, and 1% antibiotics (100 U/mL of penicillin and 100 µg/mL of streptomycin) at 37 °C in a humidified atmosphere of 5% CO_2_. Normal human astrocytes from human fetal cortex were obtained from Lonza (Walkersville, MD, USA) and cultured according to manufacturer’s instructions.

### 4.3. MTT Assay

The 3-(4, 5-dimethylthiazol-2-yl)-2,5-diphenyltetrazolium bromide (MTT, Invitrogen, Waltham, MA, USA), a colorimetric-based assay was performed to analyze the viable cells. IMR32 cells and normal human astrocytes were seeded at 2 × 10^4^ cells per well in 96-well plate in 5% CO_2_ at 37 °C. Cells were treated with 1, 10, 20, and 30 μM of neferine or 20, 50, 100, and 400 μM of TMZ for 24 h, and then 20 μL/well MTT (25 μg/mL) solution was added into the wells and further incubated for additional 3 h. The medium was removed, and formazan was solubilized by the addition of 100 μL/well dimethyl sulfoxide (DMSO, Sigma, MO, USA), and OD value was measured at 570 nm using a microplate reader (ELISA reader, Thermo Labsystems, Waltham, MA, USA). The percentage of viable cells was determined from a comparison with untreated control.

### 4.4. Cell Cycle Analysis

IMR32 cells (ATCC^®^ CCL-127™, Manassas, VA, USA) were seeded at 3 × 10^5^ cells per well in 6-well plate. Cells were treated with 10, 20, and 30 μM of neferine or 50, 100, and 400 μM of TMZ for 24 h. Cells were harvested and fixed with ice cold 70% ethanol at −20 °C overnight and washed with cold PBS twice, and then incubated in 1 mL (*v*/*v*) staining solution (20 μg/mL propidium iodide (PI), 0.1% Triton X-100, and 0.2 mg/mL RNase) at 37 °C for 30 min. Then cell were analyzed by using flow cytometer (CytomicsTM FC500, Beckman, Fullerton, CA, USA). For each experiment, 10,000 cells were counted and data were analyzed. 

### 4.5. Western Blot Analysis

IMR32 cells were seeded at 5 × 10^5^ cells per well in 6-well plate, and then incubated with 10, 20, and 30 μM of neferine or 50, 100, and 400 μM of TMZ for 24 h. After incubation, the medium was removed and the cells were washed with PBS, then cells were collected and lysed with RIPA buffer at 4 °C for 30 min and centrifuged at 12,000 × *g* for 30 min. The supernatant was collected and quantified by Bradford protein assay (Bio-Rad, Hercules, CA, USA). Protein samples (28 μg) were loaded into the well and separated with sodium dodecyl sulfate polyacrylamide gel electrophoresis (SDS-PAGE), then the gel was transferred to the PVDF membrane (Perkin Elmer Life Sciences, Boston, MA, USA). The blots were blocked with 10% non-fat milk in TBS/T (20 mM Tris-Base, 137 mM NaCl at pH 7.4 and 0.05% Tween-20) at room temperature (RT) for 30 min and then blots were incubated with the appropriate primary antibody at 4 °C overnight. The blots were washed 3 times with TBS/T, and then incubated with horseradish peroxidase (HRP)-conjugated secondary antibodies at RT for 1 h. After blots exposed to ECL reagents (PerkinElmer Life Sciences), the proteins bands were visualized and the protein expression was analyzed by Luminescent image analyzer (LAS)-3000 (Fujifilm, Minato, Tokyo, Japan).

### 4.6. Wound Healing Assay

For measuring the migration rate, IMR32 cells were seeded 3 × 10^5^ cells per well in 6-well plate. After cells reached 70–80% confluent, a 10 μL pipette tip was used to make a straight scratch for simulating a wound and then cells were treated with 10, 20, and 30 μM of neferine or 50, 100, and 400 μM of TMZ for various time points. Wound closure was monitored and photographed at 0, 8, and 16 h by ZEISS inverted microscope connected with Canon 700D camera.

### 4.7. Statistical Analysis

All the data were expressed as mean ± standard deviation (SD) of three independent experiments. Statistical comparisons of multiple variables were made by one-way analysis of variance (ANOVA) and the Tukey test. *p* value of <0.05 was considered to be statistically significant differences (* *p* < 0.05, ** *p* < 0.01, *** *p* < 0.001).

## 5. Conclusions

Together, this study demonstrates for the first time that neferine has therapeutic prospect for targeting IMR32 human neuroblastoma cells through the best fit in the binding pocket of FAK and S6K1 proteins, and caused decrease the level of these two proteins. Furthermore, neferine showed a potential effect on cell viability, induced the autophagy and apoptosis, and inhibited the cell motility of IMR32 cells. When these head-to-head comparative quality studies of drugs approved by FDA, and new drug candidate are taken into consideration, neferine could be a promising anti-brain tumor drug as clinical reagent temozolomide.

## References

[1] Maris J.M., Hogarty M.D., Bagatell R., Cohn S.L. (2007). Neuroblastoma. Lancet.

[2] Weinstein J.L., Katzenstein H.M., Cohn S.L. (2003). Advances in the diagnosis and treatment of neuroblastoma. Oncologist.

[3] Matthay K.K., Villablanca J.G., Seeger R.C., Stram D.O., Harris R.E., Ramsay N.K., Swift P., Shimada H., Black C.T., Brodeur G.M. (1999). Treatment of high-risk neuroblastoma with intensive chemotherapy, radiotherapy, autologous bone marrow transplantation, and 13-*cis*-retinoic acid. Children’s Cancer Group. New Engl. J. Med..

[4] Keshelava N., Seeger R.C., Reynolds C.P. (1997). Drug resistance in human neuroblastoma cell lines correlates with clinical therapy. Eur. J. Cancer.

[5] Rayan A., Raiyn J., Falah M. (2017). Nature is the best source of anticancer drugs: Indexing natural products for their anticancer bioactivity. PLoS ONE.

[6] Wu S., Sun C., Cao X., Zhou H., Hong Z., Pan Y. (2004). Preparative counter-current chromatography isolation of liensinine and its analogues from embryo of the seed of *Nelumbo nucifera* GAERTN. using upright coil planet centrifuge with four multilayer coils connected in series. J. Chromatogr. A.

[7] Kadioglu O., Law B.Y.K., Mok S.W.F., Xu S.W., Efferth T., Wong V.K.W. (2017). Mode of action analyses of neferine, a bisbenzylisoquinoline alkaloid of lotus (*Nelumbo nucifera*) against multidrug-resistant tumor cells. Front. Pharmacol..

[8] Poornima P., Weng C.F., Padma V.V. (2013). Neferine from *Nelumbo nucifera* induces autophagy through the inhibition of PI3K/Akt/mTOR pathway and ROS hyper generation in A549 cells. Food Chem..

[9] Ding H., Shi J.H., Wang Y., Guo J., Zhao J.H., Dong L. (2011). Neferine inhibits cultured hepatic stellate cell activation and facilitates apoptosis A possible molecular mechanism. Eur. J. Pharmacol..

[10] Selvi S.K., Vinoth A., Varadharajan T., Weng C.F., Padma V.V. (2017). Neferine augments therapeutic efficacy of cisplatin through ROS-mediated non-canonical autophagy in human lung adenocarcinoma (A549 cells). Food Chem. Toxicol..

[11] Poornima P., Kumar V.B., Weng C.F., Padma V.V. (2014). Doxorubicin induced apoptosis was potentiated by neferine in human lung adenocarcima, A549 cells. Food Chem. Toxicol..

[12] Thiyagarajan V., Lin S.H., Chang Y.C., Weng C.F. (2016). Identification of novel FAK and S6K1 dual inhibitors from natural compounds via ADMET screening and molecular docking. Biomed. Pharmacother..

[13] Scicchitano B.M., Sorrentino S., Proietti G., Lama G., Dobrowolny G., Catizone A., Binda E., Larocca L.M., Sica G. (2018). Levetiracetam enhances the temozolomide effect on glioblastoma stem cell proliferation and apoptosis. Cancer Cell Int..

[14] Wurstle S., Schneider F., Ringel F., Gempt J., Lammer F., Delbridge C., Wu W., Schlegel J. (2017). Temozolomide induces autophagy in primary and established glioblastoma cells in an EGFR independent manner. Oncol. Lett..

[15] Tomicic M.T., Meise R., Aasland D., Berte N., Kitzinger R., Kramer O.H., Kaina B., Christmann M. (2015). Apoptosis induced by temozolomide and nimustine in glioblastoma cells is supported by JNK/c-Jun-mediated induction of the BH3-only protein BIM. Oncotarget.

[16] Parsons J.T., Martin K.H., Slack J.K., Taylor J.M., Weed S.A. (2000). Focal adhesion kinase: A regulator of focal adhesion dynamics and cell movement. Oncogene.

[17] Pullen N., Thomas G. (1997). The modular phosphorylation and activation of p70s6k. FEBS Lett..

[18] Xie Z., Klionsky D.J. (2007). Autophagosome formation: Core machinery and adaptations. Nat. Cell Biol..

[19] Kang R., Zeh H.J., Lotze M.T., Tang D. (2011). The Beclin 1 network regulates autophagy and apoptosis. Cell Death Differ..

[20] Tanida I., Ueno T., Kominami E. (2008). LC3 and Autophagy. Methods in Mol. Biol..

[21] Elmore S. (2007). Apoptosis: A review of programmed cell death. Toxicol. Pathol..

[22] Friedl P., Wolf K. (2010). Plasticity of cell migration: A multiscale tuning model. J. Cell Biol..

[23] Kang J., Badger T.M., Ronis M.J., Wu X. (2010). Non-isoflavone phytochemicals in soy and their health effects. J. Agric. Food Chem..

[24] Lambert J.D., Sang S., Hong J., Yang C.S. (2010). Anticancer and anti-inflammatory effects of cysteine metabolites of the green tea polyphenol, (-)-epigallocatechin-3-gallate. J. Agric. Food Chem..

[25] Poornima P., Quency R.S., Padma V.V. (2013). Neferine induces reactive oxygen species mediated intrinsic pathway of apoptosis in HepG2 cells. Food Chem..

[26] Zhang X., Liu Z., Xu B., Sun Z., Gong Y., Shao C. (2012). Neferine, an alkaloid ingredient in lotus seed embryo, inhibits proliferation of human osteosarcoma cells by promoting p38 MAPK-mediated p21 stabilization. Eur. J. Pharmacol..

[27] Senderowicz A.M., Sausville E.A. (2000). Preclinical and clinical development of cyclin-dependent kinase modulators. J. Natl. Cancer Inst..

[28] Simmons Kovacs L.A., Orlando D.A., Haase S.B. (2008). Transcription networks and cyclin/CDKs: The yin and yang of cell cycle oscillators. Cell Cycle.

[29] Gloria N.F., Soares N., Brand C., Oliveira F.L., Borojevic R., Teodoro A.J. (2014). Lycopene and beta-carotene induce cell-cycle arrest and apoptosis in human breast cancer cell lines. Anticancer Res..

[30] Huang W.S., Kuo Y.H., Kuo H.C., Hsieh M.C., Huang C.Y., Lee K.C., Lee K.F., Shen C.H., Tung S.Y., Teng C.C. (2017). CIL-102-Induced Cell Cycle Arrest and Apoptosis in Colorectal Cancer Cells via Upregulation of p21 and GADD45. PLoS ONE.

[31] Ji L., Zhong B., Jiang X., Mao F., Liu G., Song B., Wang C.Y., Jiao Y., Wang J.P., Xu Z.B. (2017). Actein induces autophagy and apoptosis in human bladder cancer by potentiating ROS/JNK and inhibiting AKT pathways. Oncotarget.

[32] Shin S.Y., Yong Y., Kim C.G., Lee Y.H., Lim Y. (2010). Deoxypodophyllotoxin induces G2/M cell cycle arrest and apoptosis in HeLa cells. Cancer Lett..

[33] Zhang L., Zheng Y., Deng H., Liang L., Peng J. (2014). Aloperine induces G2/M phase cell cycle arrest and apoptosis in HCT116 human colon cancer cells. Int. J. Mol. Med..

[34] Golubovskaya V.M., Cance W.G. (2007). Focal adhesion kinase and p53 signaling in cancer cells. Int. Rev. Cytol..

[35] Zhao J.H., Bian Z.C., Yee K., Chen B.P.C., Chien S., Guan J.L. (2003). Identification of transcription factor KLF8 as a downstream target of focal adhesion kinase in its regulation of cyclin D1 and cell cycle progression. Mol. Cell.

[36] Ding Q., Grammer J.R., Nelson M.A., Guan J.L., Stewart J.E., Gladson C.L. (2005). p27Kip1 and cyclin D1 are necessary for focal adhesion kinase regulation of cell cycle progression in glioblastoma cells propagated in vitro and in vivo in the scid mouse brain. J. Biol. Chem..

[37] Feng R., Yang S. (2016). Effects of combining erlotinib and RNA-interfered downregulation of focal adhesion kinase expression on gastric cancer. J. Int. Med. Res..

[38] Shi R., Wang Q., Ouyang Y., Wang Q., Xiong X. (2016). Picfeltarraenin IA inhibits lipopolysaccharide-induced inflammatory cytokine production by the nuclear factor-kappaB pathway in human pulmonary epithelial A549 cells. Oncol. Lett..

[39] Woo M.S., Ohta Y., Rabinovitz I., Stossel T.P., Blenis J. (2004). Ribosomal S6 kinase (RSK) regulates phosphorylation of filamin a on an important regulatory site. Mol. Cell. Biol..

[40] Ismail H.M. (2012). Overexpression of s6 kinase 1 in brain tumours is associated with induction of hypoxia-responsive genes and predicts patients’ survival. J. Oncol..

[41] Sinclair C.S., Rowley M., Naderi A., Couch F.J. (2003). The 17q23 amplicon and breast cancer. Breast Cancer Res. Treat..

[42] Sivalingam K.S., Paramasivan P., Weng C.F., Viswanadha V.P. (2017). Neferine Potentiates the Antitumor Effect of Cisplatin in Human Lung Adenocarcinoma Cells Via a Mitochondria-Mediated Apoptosis Pathway. J. Cell. Biochem..

[43] Liu Y.L., Yang P.M., Shun C.T., Wu M.S., Weng J.R., Chen C.C. (2010). Autophagy potentiates the anti-cancer effects of the histone deacetylase inhibitors in hepatocellular carcinoma. Autophagy.

[44] Mizushima N., Yoshimori T., Ohsumi Y. (2011). The role of Atg proteins in autophagosome formation. Ann. Rev. Cell Dev. Biol..

[45] Cao Q.H., Liu F., Yang Z.L., Fu X.H., Yang Z.H., Liu Q., Wang L., Wan X.B., Fan X.J. (2016). Prognostic value of autophagy related proteins ULK1, Beclin 1, ATG3, ATG5, ATG7, ATG9, ATG10, ATG12, LC3B and p62/SQSTM1 in gastric cancer. Am. J. Transl. Res..

[46] Li W.L., Xiong L.X., Shi X.Y., Xiao L., Qi G.Y., Meng C. (2016). IKKbeta/NFkappaBp65 activated by interleukin-13 targets the autophagy-related genes LC3B and beclin 1 in fibroblasts co-cultured with breast cancer cells. Exp. Ther. Med..

[47] Shin J.Y., Hong S.H., Kang B., Minai-Tehrani A., Cho M.H. (2013). Overexpression of beclin1 induced autophagy and apoptosis in lungs of K-rasLA1 mice. Lung Cancer.

[48] Nascimento-Ferreira I., Santos-Ferreira T., Sousa-Ferreira L., Auregan G., Onofre I., Alves S., Dufour N., Colomer Gould V.F., Koeppen A., Deglon N. (2011). Overexpression of the autophagic beclin-1 protein clears mutant ataxin-3 and alleviates Machado-Joseph disease. Brain.

[49] Cheng Z., Zhu Q., Dee R., Opheim Z., Mack C.P., Cyr D.M., Taylor J.M. (2017). Focal Adhesion Kinase-mediated Phosphorylation of Beclin1 Protein Suppresses Cardiomyocyte Autophagy and Initiates Hypertrophic Growth. J. Biol. Chem..

[50] Philchenkov A., Zavelevich M., Kroczak T.J., Los M. (2004). Caspases and cancer: Mechanisms of inactivation and new treatment modalities. Exp. Oncol..

[51] Slee E.A., Adrain C., Martin S.J. (2001). Executioner caspase-3, -6, and -7 perform distinct, non-redundant roles during the demolition phase of apoptosis. J. Biol. Chem..

[52] Yang Y., Zhao S., Song J. (2004). Caspase-dependent apoptosis and -independent poly(ADP-ribose) polymerase cleavage induced by transforming growth factor beta1. Int. J. Biochem. Cell Biol..

[53] Cheng Z., DiMichele L.A., Rojas M., Vaziri C., Mack C.P., Taylor J.M. (2014). Focal adhesion kinase antagonizes doxorubicin cardiotoxicity via p21(Cip1.). J. Mol. Cell. Cardiol..

[54] Cheng Z., DiMichele L.A., Hakim Z.S., Rojas M., Mack C.P., Taylor J.M. (2012). Targeted focal adhesion kinase activation in cardiomyocytes protects the heart from ischemia/reperfusion injury. Arterioscler. Thromb. Vasc. Biol..

[55] Thomas S.A., Thamkachy R., Ashokan B., Komalam R.J., Sreerekha K.V., Bharathan A., Santhoshkumar T.R., Rajasekharan K.N., Sengupta S. (2012). Diaminothiazoles inhibit angiogenesis efficiently by suppressing Akt phosphorylation. J. Pharmacol. Exp. Ther..

